# The Impact of Functional Bars and Adapted Physical Activity on Quality of Life in Chronic Kidney Disease: A Pilot Study

**DOI:** 10.3390/ijerph19063281

**Published:** 2022-03-10

**Authors:** Elisa Grazioli, Eliana Tranchita, Giulia Marrone, Silvia Urciuoli, Manuela Di Lauro, Claudia Cerulli, Nicolò Piacentini, Arianna Murri, Roberto Celotto, Annalisa Romani, Attilio Parisi, Nicola Di Daniele, Annalisa Noce

**Affiliations:** 1 Department of Exercise, Human and Health Sciences, Foro Italico University of Rome, 00135 Rome, Italy; elisa.grazioli@uniroma4.it (E.G.); eliana.tranchita@gmail.com (E.T.); claudia.cerulli@uniroma4.it (C.C.); ariannamurri@hotmail.it (A.M.); attilio.parisi@uniroma4.it (A.P.); 2 Department of Experimental and Clinical Medicine, “Magna Graecia” University, 88100 Catanzaro, Italy; 3 UOC of Internal Medicine—Center of Hypertension and Nephrology Unit, Department of Systems Medicine, University of Rome Tor Vergata, Via Montpellier 1, 00133 Rome, Italy; manuela.dilauro@alumni.uniroma2.eu (M.D.L.); npiacentini@yahoo.it (N.P.); didaniele@med.uniroma2.it (N.D.D.); 4 PHYTOLAB (Pharmaceutical, Cosmetic, Food Supplement, Technology and Analysis), DiSIA, University of Florence, Via Ugo Schiff 6, Sesto Fiorentino, 50019 Florence, Italy; silvia.urciuoli@gmail.com (S.U.); annalisa.romani@unfi.it (A.R.); 5 Department of Cardiovascular Disease, Tor Vergata University of Rome, 00133 Rome, Italy; roberto.celotto@alumni.uniroma2.eu

**Keywords:** chronic kidney disease, adapted physical activity, natural bioactive compounds, uremic sarcopenia, polyphenols

## Abstract

Chronic kidney disease (CKD) represents a public health problem because it is characterized by several comorbidities, including uremic sarcopenia (US), and a poor quality of life. Currently, there are no standardized treatments available to counteract the onset of US but only some possible therapeutic approaches to slow its progression. The aim of this pilot study is to collect descriptive data in order to design a clinical trial based on the power analysis and simple size. The purpose of this pilot study was to evaluate the possible beneficial action induced by the functional anti-inflammatory and antioxidant bars in combination with the adapted physical activity (APA), on the onset and progression of US and other related-CKD comorbidities. We enrolled 21 CKD patients under conservative therapy, divided into four groups: (A) the physical exercise program (PEP), three times a week, in combination with the daily consumption of the two functional bars group; (B) the PEP group; (C) the daily consumption of the two functional bars group; (D) the control group. The duration of the study protocol was 12 weeks. We observed an improvement trend of body composition, blood pressure levels, lipid metabolism, and functional test in A and B groups. These preliminary data would seem to confirm the effectiveness of APA and to demonstrate the additive role of the natural bioactive compound’s assumption in countering US and other CKD comorbidities.

## 1. Introduction

Chronic kidney disease (CKD) has become a significant and increasing problem for world public health in the last years, as it strongly impacts on global morbidity and mortality and because it represents an important cardiovascular (CV) risk factor [1]. After SARS-CoV-2 pandemic, CKD continued to be a major health problem. In fact, most of the patients who died from COVID-19 had one or more comorbidities, and specifically 20% of them presented CKD [2].

CKD is characterized by a series of comorbidities that significantly impact on the quality of life (QOL) of nephropathic patients [3]. Among these comorbidities, CV diseases and uremic sarcopenia (US) play a leading role in raising mortality [4] and in worsening the functional autonomy of patients, increasing the risk of fall and fractures and the need for hospitalization [5,6,7,8].

Sarcopenia, according to the updated guidelines of the European Working Group on Sarcopenia in Older People (EWGSOP2), drawn up in 2019, is defined as a decrease in lean mass and muscle strength, in association or not with a decline in physical performance, a parameter used to stage the severity of the pathology [9,10].

In CKD patients, the sarcopenia is particularly frequent, and it is triggered by hormonal and nutritional disorders, alterations of peripheral oxygenation, metabolic acidosis, electrolytes alterations, physical inactivity, and alteration of the composition of muscle fibers [11,12]. Various risk and predisposing factors that interact and amplify each other contribute and concur to the pathogenesis of US: (i) vitamin D deficiency, (ii) overactivation of the ubiquitin-proteasome pathway, (iii) insulin resistance, (iv) chronic low-grade inflammatory state, (v) activation of angiotensin II, (vi) depletion of carnitine reserves, and (vii) alterations in appetite mechanism [11,13]. Regarding the ubiquitin-proteasome pathway (UPP), it is activated as result of metabolic acidosis and it, in turn, stimulates the intracellular protein degradation [14]. 

Insulin resistance is often associated with CKD, and its incidence increases linearly with the decline in renal function [14]. In response to insulin resistance, the body uses less glucose as an energy source, as to stimulate hepatic gluconeogenesis and to decrease glucose uptake by the muscle and the liver.

CKD induces, from the early stages, a chronic low-grade inflammatory state, which can be evaluated by monitoring its typical biomarkers, such as C-reactive protein (CRP), interleukin-6 (IL-6), and tumor necrosis factor-α (TNF-α) [11]. In particular, TNF-α appears to play a pivotal role in the activation of catabolic processes by increasing caspase-3 activity and inducing insulin resistance [13].

In CKD patients, angiotensin II is activated by muscle catabolism, and it is able to further increase proteolysis, reducing blood levels of insulin-like growth factor-1 (IGF-1) and activating the transforming growth factor-β (TGF-β) pathway, which leads to the loss of muscle mass [15]. It has also been shown that increased angiotensin II production can reduce the reserve of satellite cells, altering muscle regeneration [16].

CKD patients show a decrease in appetite due to various factors, including physical inactivity, high levels of uremic toxins, chronic inflammatory status, and underlying hormonal disorders, such as reduced production of ghrelin and neuropeptide Y and increased leptin levels, which stimulates satiety and mediates inflammation [13,17,18].

To date, there are no specific and standardized treatments available to counteract the onset of US but only some possible therapeutic approaches to slow its progression, such as adapted physical activity (APA), nutritional supplements, drugs to counteract metabolic acidosis, and insulin resistance. In particular, the cornerstones of the treatment seem to be diet-nutritional therapy and physical exercise, two approaches without relevant side effects [11,19]. Among the natural bioactive compounds, polyphenols seem to play an important role in improving the state of health in chronic degenerative non-communicable diseases (CDNCDs) patients. Among these, CKD deserves a relevant mention, as described above [20,21,22,23,24,25].

In CKD patients, not only in conservative therapy but also in renal replacement therapy (RRT), physical exercise is recommended due to its positive influence on VO_2_max, muscle strength and muscle mass, physical performance, self-reported physical function, and QOL [26,27]. According to the latest evidence, recommendations for the practice of physical activity (PA) for CKD patients support exercising regularly for >30 min/session at least three times a week, including aerobic, strength and flexibility exercises [28]. Most of the studies have been conducted on aerobic training [29], few data are available on resistance training, and it is unclear how this type of PA can influence catabolic/inflammatory processes typical of CKD. However, strength exercises seem to induce beneficial effects on bone density and muscle mass in these patients, especially in RRT [30].

The aim of this pilot study is to collect descriptive data in order to design a clinical trial based on the power analysis and sample size. The purpose of this pilot study was to evaluate the possible beneficial action induced by the combination of the functional bars assumption, characterized by high anti-inflammatory and antioxidant activity, with the APA, on the onset and progression of US and other related-CKD comorbidities. In particular, we have examined the possible benefits induced by the two therapeutic approaches on body composition, oxidative stress, muscle strength, physical performance, and other comorbidities and metabolic dysfunction (such as lipid, purine and glucose metabolism) [31,32,33].

## 2. Materials and Methods

### 2.1. Patients

At the Hypertension Unit and Nephrology Centre of the University Hospital of Rome Tor Vergata, we enrolled 21 CKD patients under conservative therapy (stage I-IIIb according to the kidney disease: improving global outcomes (KDIGO) guidelines [34], 10 males and 11 females (mean age 62.7 ± 5.0 years). The study protocol lasted twelve weeks. Inclusion criteria were: subjects with CKD stage I-IIIb, according to KDIGO guidelines [34], with age comprised between 55-70 years. Exclusion criteria were: cancer in the active phase, HIV+, HBsAg+ HCV+, refusal to sign informed consent, non-obtaining the eligibility for the practice of recreational-motor activity, inflammatory and infectious diseases in the acute phase, presence of hyperkalemia. All enrolled patients gave their informed consent before starting the study. The experimental protocol complies with the 1975 guidelines of the Declaration of Helsinki and it has been approved by the Independent Ethics Committee of the University Hospital of Rome Tor Vergata. The protocol code is 223/20 of 7 December 2020.

The study population was divided into 4 subgroups (A, B, C, and D). In particular, group A carried out the physical exercise program online at home, three times a week in combination with the daily consumption of the two functional bars; group B carried out only the physical exercise program online at home three times a week; group C daily consumed the two functional bars; group D represented the control group.

**Figure 1:**
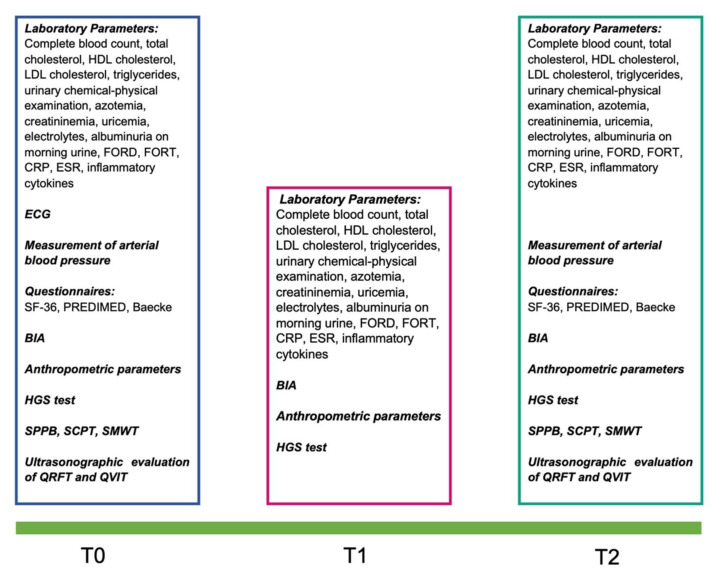
Laboratory parameters, tests, and questionnaires performed at the various times of the study. Abbreviations: BIA, bioelectrical impedance analysis; CRP, C-reactive protein; ECG, electrocardiogram; ESR, erythrocyte sedimentation rate; FORD, free oxygen radical defense; FORT, free oxygen radicals test; HDL, high-density lipoproteins; HGS-test, handgrip strength-test; LDL, low-density lipoproteins; PREDIMED, prevención con dieta Mediterránea; QRFT, quadriceps rectus femoris thickness; QRFT, quadriceps vastus intermedius thickness; SF-36, short form-36; SCPT, stair climb power test; SMWT, six-minutes walking test; SPPB, short physical performance battery.

At the time of the enrolment (T0) and in subsequent controls, namely after six weeks (T1) and after twelve weeks (T2), patients underwent the following evaluations, as reported in Figure 1.

### 2.2. Laboratory Parameters

Routine blood and urinary parameters were monitored in each time point of the study. In particular, we evaluated azotemia, creatinine, uric acid, electrolytes (such as potassium, phosphorus, sodium and calcium), sideremia, glycemia, lipid profile, chemical–physical urinary examination, and albuminuria on morning urine. Moreover, inflammatory parameters such as CRP, erythrocyte sedimentation rate (ESR), IL-6 and TNF-α were also evaluated. All laboratory parameters were analyzed by Dimension Vista 1500 (Siemens Healthcare Diagnostics, Milano, Italy), while the lipid profile was detected by standard enzymatic colorimetric techniques (Roche Modular P800, Roche Diagnostics, Indianapolis, IN, USA). The IL-6 and TNF-α were monitored by IMMULITE 2000 XPi Immunoassay System (Siemens Healthcare Diagnostics, Milano, Italy). All parameters were analyzed according to standard procedures in the Clinical Chemistry Laboratories of the University Hospital of Rome Tor Vergata.

All patients also underwent capillary sampling using CR4000 tool for the oxidative stress evaluation and for the assessment of antioxidant defense mechanisms. In particular, the free oxygen radical test (FORT) and the free oxygen radical defense (FORD) were performed. The former test measures the levels of circulating oxygen, free radicals, and the second one indirectly determines the organisms’ antioxidant capacity by measuring the concentration of ascorbic acid, glutathione, and albumin [35,36].

### 2.3. Questionnaires

At T0 and at T2 (after twelve weeks), Prevención con Dieta Mediterránea (PREDIMED), short form-36 (SF-36), and Baecke questionnaires were administrated to assess, respectively, adherence to the Mediterranean diet, patient’s QOL and the baseline levels of PA. All the listed questionnaires were administered face to face with patients, at all-time points of the study. 

The PREDIMED questionnaire was able to assess through 14 items the adherence to Mediterranean diet (MD). In fact, each item corresponded to 1 point up to a maximum of 14 points. According to the scoring, patients should be divided into 3 MD adherence groups: minimal adherence (5 or less points), medium adherence (points comprised between 6 and 9), maximum adherence (more o equal to 10 points) [37,38]. 

The SF-36 is a questionnaire that assessed the patient’s QOL through a visual score. It is composed of 36 items that investigate 9 symptoms spheres: physical functioning, role limitations due to physical health, role limitations due to emotional problems, energy/fatigue, emotional well-being, social functioning, pain, general health and, health change [39]. For each sphere, it is possible to obtain a value comprised between 0 and 100 points referred to each sphere, that are directly proportional to psychophysics well-being. 

Finally, the Baecke questionnaire evaluated the subject’s PA degree in three spheres: job, sport, and other free time activities [40].

### 2.4. Body Composition Assessment

Patient’s anthropometric parameters were detected. Body weight (kg) was measured to the nearest 0.01 kg using a balance scale (Seca 711, Hamburg, Germany). Height (m) was measured using a stadiometer to the nearest 0.1 cm (Seca 220, Hamburg, Germany). Body mass index (BMI) was calculated as body weight divided by height squared (kg/m^2^). For the assessment of body composition, all nephropathic patients underwent bioelectrical impedance analysis (BIA). Resistance, reactance, impedance, and phase angle at 50 kHz frequency were measured using a BIA 101S instruments (Akern/RIL System-Florence). 

For the evaluation of body composition we considered the following parameters: total body water (TBW), extracellular water (ECW), body cell mass (BCM), fat free mass (FFM) and, fat mass (FM) [41,42]. 

### 2.5. Ultrasound Evaluation

At T0 and at T2 (after twelve weeks), the patients underwent ultrasound evaluation of *quadriceps rectus femoris thickness* (QRFT) and *quadriceps vastus intermedius thickness* (QVIT) as this ultrasound approach seemed useful in the detection of muscle loss in CKD patients [43,44]. This ultrasonographic evaluation was carried out by B-mode modulation with a 7.5 MHz transducer. The probe was placed perpendicular to the long axis of the muscle, which was covered with an abundant gel layer; minimal external pressure was exerted in order to prevent muscle compression. Three measurements were made bilaterally, in the supine position, with both knees in extension, at the level of two standardized points: the midpoint between the anterosuperior iliac spine and the upper limit of the patella and the boundary point between the lower third and the upper two-thirds of the quadriceps muscle. The values obtained were compared with the average of the measures evaluated in the reference population, as suggested by Sabatino et al. [45,46,47]. All ultrasounds were carried out by the same healthcare professional (A.N.) with an ultrasonographic experience of 15 years in order to reduce the bias related to intra-operator variability and with the same ultrasound equipment (Esaote MyLab70 XVision) with linear probe LA523.

### 2.6. Evaluation of Muscle Strength and Physical Performance

For the assessment of muscle strength, we used the *handgrip strength* (HGS) test, a dynamometer that evaluates the handgrip force (Jamar Plus). The cut-offs of HGS are <30 kg for men and <20 kg for women [9]. The patient performed the test in a sitting position with a 90-degree flexed elbow; the researcher set up the instrument and asked the patient to squeeze as hard as possible for a few seconds. Three repetitions *per* side were performed, considering the average value.

To assess physical performance, patients executed the *short physical performance battery* (SPPB), the *stair climb power test* (SCPT), and the *six-minute walk test* (SMWT), which measure balance, gait speed, and dynamic elongation [48,49]. The SPPB is a combination of 3 tests assessing gait speed (4 m walking), power (repeated chair stand), and balance (tandem test). Each test is scored out of 4 and their sum indicates the level of performance where 12 is the best score. The SCPT analyses the power of the lower limb where the participant has to climb 10 steps as fast as possible (no running or jumping). The time taken to complete the task was recorded. The SMWT evaluates the functional capacity. Patients have to walk as far as possible (no running or jumping) for 6 min on a flat corridor and the researcher records the walking distance. At the end of the test, the fatigue sensation was evaluated through the BORG Scale (0–10). 

### 2.7. Other Functional Parameters

At the beginning of the study (T0), all patients underwent a medical examination carried out by a specialist in Sports Medicine, including the anamnesis, a 12 leads electrocardiogram (ECG) at rest, and monitoring of systemic arterial pressure, in order to obtain the eligibility for the practice of recreational-motor activity and to exclude possible complications related to the training.

### 2.8. Composition of the Bars

The composition of the two functional organic bars, weighing 32 g each, with a caloric intake of 122 kcal and average potassium content of 200 mg *per* bar, is shown in Table 1. 

The two bars selected for the study are based on organic fruit and vegetables, and they were added with secondary raw materials from the wine and oil supply chain. In particular, grape micronized pomace and grape seeds, and olive leaf powder were chosen for their content in antioxidant compounds, as reported in the study by our research group concerning the innovative production of these functional ingredients and their related chemical characterization made by high-performance liquid chromatography–diode array detector–mass spectrometry (HPLC-DAD-MS) [50]. Regarding the ingredients derived by *Olea europaea* L., the organic extra virgin olive oil (EVOO) was present in both bars, while the organic olive leaf powder was present only in bar 2. These two products were selected for their high content in minor polar compounds (MPCs) and for their biological activities linked to molecules such as hydroxytyrosol, oleuropein aglycone, oleuropein, oleocanthal, oleacin, and verbascoside [23,51,52,53]. Regarding the ingredients derived by *Vitis vinifera* L. (micronized grape pomace and grape seeds), they come from grapes rich in antioxidant compounds such as proanthocyanidins, anthocyanins (cyanidin, malvidin, peonidin) and flavonols (mainly quercetin and its derivatives) [50].

Two parameters of the two functional organic bars were evaluated: the total antioxidant capacity by Folin–Coicalteu analysis, expressed in mg of gallic acid (GAE)/32 g, and the percentage antiradical activity (% AA) using the DPPH radical method [54]. The results of the analyzes relating to the total antioxidant capacity show that, for bar 1, it was 209.54 mgGAE/32 g and, for bar 2, it was 155.52 mgGAE/32 g. The results relating to % AA show that bar 1 had 84.49% and bar 2 had 80.53%. 

### 2.9. Physical Exercise Protocol

Patients randomly placed in groups A or B followed an online and supervised combined PA protocol, with an aerobic phase and a strength-focused phase lasting about 60 min, performed at home, three times a week. The training session was carried out entirely online, through video lessons, under the supervision of a Graduate Specialized in Motor Sciences at the University of Rome Foro Italico. In detail, the tailored exercise protocol included a general warm-up lasting about 15 min, with exercises aimed at improving joint mobility and balance. The central phase, lasting about 30–35 min, involved 3 sets composed by 3 strength exercises for both upper and lower limbs using elastic bands and an aerobic activity at ≃65–70% of patient heart rate reserve, evaluated through the Karvonen formula. Regarding the strength part: during the first four weeks the trainer proposed exercise for the anterior/posterior chain, where patients performed 2 sets of 2 circuits composed by 4 multi-joint exercises, bodyweight or using the resistance band Core training, which included exercises aimed to improve abdominal, lumbar, and pelvis muscle strength, was incorporated in the session, as well. In the last eight weeks, exercises were focused on the upper/lower chain, patients started with 2 sets of 4 exercises during the weeks 4–8, then in weeks 8–12, they performed 3 sets of 3 exercises. The last part of the protocol, lasting 10 min, focused on cooling down and stretching of the main muscle groups involved during training, such as abdominal, quadriceps, pectoralis muscles, etc. At the end of each phase, patients’ heart rate and perceived fatigue were monitored by the trainer using patients’ personal heart rate monitor and the Borg Scale. All patients completed the protocol and no adverse events related to exercise were experienced.

### 2.10. Statistical Analysis

All data were entered into an Excel spreadsheet (Microsoft, Redmond, WA, USA) and the analysis was performed using the Windows Social Science Statistics Package, version 25.0 (IBM_SPSS, Chicago, IL, USA).

The descriptive statistics considered the mean ±standard deviation for the parameters with normal distribution (after confirmation with histograms and the Shapiro–Wilk test), while for the non-normal variables, it was considered the median and the interval (minimum–maximum). 

The primary aim of the study was to assess the descriptive statistic of the size, which concerned the central tendency, variability, symmetry, and kurtosis. The comparison for the non-normal variables were obtained with the Pearson’s first skewness. The aim of this statically evaluation was to set the power analysis (sample size) for a subsequent randomized clinical trial and to estimate the effects size.

## 3. Results

Currently, a total of 21 CKD patients were recruited in this study. Patients were divided in four subgroups (Group A, 6; Group B, 5; Group C, 5; Group D, 5). Laboratory and body composition parameters as well as physical performance tests were assessed at T0 (baseline) and T1 (after 12 weeks).

In Table 2 are reported the anthropometric and epidemiological features of the study groups at the T0.

At time of enrolment (T0), eight patients (two from group A, three from group B, two from group C and one from group D) presented a pre-sarcopenia condition, due to a decrease in the musculoskeletal mass index (SMI) obtained by BIA [55,56] or a decrease of muscle strength, evaluated by HGS test, below the reference values of the general population matched for gender and age [56].

The main laboratory findings are resumed in Table 3. At T1, we observed a significant increase in creatinine and azotemia values in Group D, control group, respectively 0.96 (0.95–1.32) mg/dL vs. 1.23 (1.09–1.31) mg/dL and 56 (56–80) mg/dL vs. 79 (76–88) mg/dL. On the contrary, total cholesterol values showed a decrease in intervention groups A and B: Group A 196 (188–240) mg/dL vs. 170 (153–215) mg/dL; Group B 245 (230–333) mg/dL vs. 200 (195–234) mg/dL, but no relevant changes were detected in groups C and D (control group). Other laboratory parameters did not show any considerable difference between T0 and T1, as shown in Table 3.

Blood pressure parameters showed a reduction for diastolic blood pressure in Group A (89; 77–118 mmHg) vs. (72; 65–95 mmHg) and Group B (89; 82–100 mmHg) vs. (80; 64–85 mmHg). Systolic blood pressure did not show any relevant difference (Table 4).

Inflammation and oxidative stress parameters are reported in Table 5 and did not show any relevant difference in the two times of the study (T0 vs. T1). 

Body composition parameters are reported in Table 6. At T1, we observed a reduction of resistance in Group B (569; 525–649 Ω vs. 484; 442–552 Ω), of reactance in Group A (38; 31–45 Ω vs. 45; 37–62 Ω) and Group B (51; 40–58 Ω vs. 60; 56–68 Ω), of phase angle in Group A (4.7; 3.8–5.0° vs. 5.9; 5.0–6.5)°, Group B (5.5; 5.1–5.8° vs. 6.1; 5.6–6.6°) and Group C (4.2; 3.8–5.1° vs. 5.1; 4.5–6.0°). ECW% showed a reduction at T1 in Group A (51.6; 49.1–58.5% vs. 46.5; 52.5–43.6%) and Group B (48.0; 46.7–50.3% vs. 45.4; 42.9–46.1%). FM% has decreased in Group A (34.6; 23.1–38.7% vs. 28.3; 18.8–30.2%) and in Group B (36.0; 24.1–37.0% vs. 25.6; 18.4–37.8%). In addition, FFM% was higher at T1 in Group A (64.6; 61.9–65.6% vs. 74.7; 68.3–81.2%) and Group B (65.7; 62.5–74.3% vs. 77.6; 66.2–82.6%). As for the US evaluation, no relevant changes were observed within each group of patients at the end of the protocol. However, we observed a trend towards a general increase in the thickness of the QVIT, particularly in treatment groups A, B, and C.

**Figure 2:**
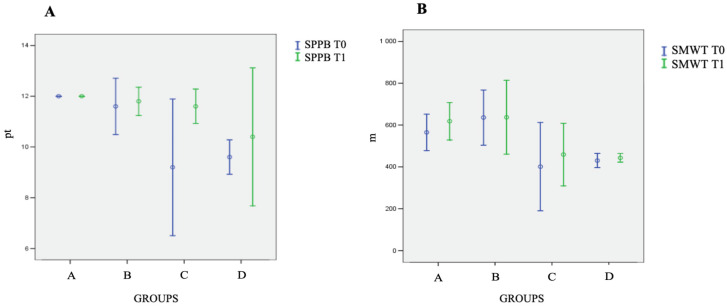
Results of the SPPB (**A**) and SMWT (**B**) tests in the various groups. Abbreviations: pt, points; SPPB, short physical performance battery; SMWT, six-minute walking test.

As it is reported in Figure 2, functional evaluations showed an improved SPPB in Group D (9.2; 6.2–11.8 points vs. 11.6; 10.5–12.1 points), and an increased walking distance, evaluated through the SMWT, in Group A (585.0; 474.8–640.2 m vs. 630.0; 490.0–695.4 m), and in Group C (401.0; 188.7–600.2 m vs. 459.0; 310.3–596.9 m).

## 4. Discussion

In our study, we observed a positive impact of PA on lipid metabolism. In particular, total cholesterol showed a reduction in groups A and B, confirming how PA alone should be useful in ameliorating lipid metabolism and highlighting, for the first time, that physical exercise in combination with the assumption of functional bars improves the lipid profile in CKD patients [57,58]. The beneficial effect induced by PA on lipid metabolism has already been described in literature [59], demonstrating how an appropriate physical exercise, both of low and moderate intensity, induces a significant reduction of total cholesterol. As suggested in the literature, PA seems to enhance the capacity of skeletal muscles to utilize lipids instead of glycogen, favoring the reduction of plasma lipids. Recent guidelines suggest that in patients with elevated levels of low-density lipoprotein (LDL) cholesterol and triglycerides but with limited mobility due to other pathologies, it is important to increase aerobic PA as much as is feasible and to incorporate resistance training in major muscle groups to improve the lipid profile [60]. Moreover, the dose–response relationship between the lipid profile and energy expenditure seem to be regardless of exercise modality. Energy expenditure associated with aerobic exercise have been shown to influence lipid profile positively. In fact, the addition of resistance training to aerobic exercise enhances the beneficial effects of PA on the lipid profile [60].

In group C patients, we did not observe a trend of reduction of total cholesterol, probably due to the lower baseline levels of total cholesterol in these patients compared to other study groups.

Moreover, the data obtained from our study showed a relevant increase in azotemia and creatinine values in the control group (group D), compared to other groups. Probably, these negative data are related to the natural progression of CKD.

In the literature, it is well-known that a higher level of arterial blood pressure is a factor related to the faster progression of CKD to end-stage renal disease (ESRD) [61]. Furthermore, as previously described in the literature, PA induces a positive impact on systemic blood pressure values, both in CKD patients [62] and in type II diabetes mellitus patients [63]. Many studies in the literature show that different modalities of exercise training produce similar and consistent improvement in arterial blood pressure. At the beginning, it was demonstrated that only aerobic exercise was able to reduce arterial blood pressure, but recently, both resistance training and combined training seem to be able to reduce blood pressure, as well [64]. All the modalities of exercise are able to produce positive effects in endothelium-dependent vasodilation. Although the stimuli may be different, both aerobic exercise and resistance training appear to induce similar benefits to the endothelium. Aerobic exercise causes repeated perfusion that leads to higher shear stress in the vascular wall. While during resistance training, the mechanical compression of resistance vessels, induced by muscle contractions, produces at first a transient ischemia, then, when muscles relax, the release of blood flow produces local hyperemia and the subsequent increase in shear stress [64].

In our study, we observed a reduction in diastolic blood pressure and in ECW% in patients of groups A and B, speculating that the combination of APA with the intake of functional bars should inhibit the renin–angiotensin–aldosterone system (RAAS) [65,66,67,68,69]. In fact, among the constituents of the bars, characterized by a high content of polyphenols, there are micronized grape pomace, grape seeds and organic olive leaf powder extracts. The latter shows an antihypertensive action related to the possible inhibition of renin production and activity [70,71]. The results of the present study are in agreement with those of a previous clinical trial conducted by Noce et al., which highlighted, in a population of male nephropathic patients, an improvement in blood pressure values, after 5 weeks of administration of an oral food supplement characterized by a high antioxidant and anti-radical activity [22].

Regarding the body composition assessment, in groups A, B, and C, we observed a substantial increase in phase angle values. Phase angle is a BIA-measured parameter that reflects the integrity of cell membranes [72]. The reduction of this parameter, as widely described in the literature, seems to correlate, in ESRD patients, with increased mortality for all causes [72,73,74]. This positive effect seems to be particularly relevant in Group A, where the combination of APA with the assumption of functional bars seems to exert an additive effect on the decrease of mortality.

Moreover, BIA showed a decrease of FM% in groups A and B and an increase of FFM% in the same groups.

Some of these data could be explained by considering the composition of the functional bars administered in this study. As previously mentioned, the bars are based on EVOO with a high content of active MPCs with antioxidant power. Some studies have shown how the intake of EVOO rich in MPCs, in particular hydroxytyrosol, can exert positive effects on health, including the reduction of total cholesterol and the protection of blood lipids from oxidative stress, as also described from the health claim of European food safety authority (EFSA) EU 432/2012 [75,76,77]. The use of EVOO, as the main source of lipids for the formulation of functional bars, could partially explain the results obtained in relation to the modulation of diastolic blood pressure. The EVOO assumption seems to reduce the ROS production that triggers the activation of RAAS. Moreover, in an animal study on spontaneously hypertensive rats, both arterial blood pressure and vascular expression of transcription factors (such as nuclear factor kappa-light-chain-enhancer of activated B cells-NF-κB) were downregulated by antioxidant compounds present in EVOO [78,79]. 

The presence of micronized *Vitis vinifera* L. also explains the improvement of some parameters examined in our study. In particular, the proanthocyanidins have shown beneficial effects on health, including the reduction of total cholesterol and of blood pressure and exerting cardiovascular protection [80,81].

In this study, as already demonstrated in previous studies by our research group, the intake of products based on fruit, vegetables, EVOO, and natural extracts rich in antioxidant compounds belonging to the class of polyphenols, showed an improvement in the clinical status of CKD patients [20,21,22,23,25]. The obtained results confirm the positive effect of APA on body composition and on clinical status, alone or in synergy with functional bars, resulting in an adjuvant tool to counteract related-CKD comorbidities [11,82].

## 5. Conclusions

Our preliminary data indicate the healthy effect of APA in CKD patients. For the first time, we evidenced the possible additive role of natural bioactive compounds with high antioxidant and anti-inflammatory action on the slowing and on the management of related-CKD comorbidities, in addition to APA. In fact, these two innovative approaches, free from side effects, seem to empower each other, ameliorating the metabolic alterations due to CKD itself. In particular, the body composition improvements characterized by an increased phase angle and FFM% and a decreased FM% and ECW%, seem to be encouraging. The data obtained in this pilot study need to be confirmed by a randomized clinical trial, conducted on a larger population.

## Figures and Tables

**Table 1:** Composition of functional bars based on organic fruit and vegetables. Legend: * Organic.

**Name**	**Ingredients**	**Weight (g)**
**Bar 1**	dates *, thompson grapes *, cashews nuts *, raw cocoa butter *, extra virgin olive oil * 5.7% almonds *, plums * 5%, kiwi powder * 3%, micronized grape pomace * 2.8%, carob flour * 2.5%, acerola powder * 2%, cabbage powder * 2%, beet powder * 2%, micronized grape seeds * 1.4%, açai powder * 1%, blueberry powder * 1%, rhubarb powder * 0.5%.	32
**Bar 2**	dates *, cashews nuts *, thompson grapes *, raw cocoa butter *, figs * 9.6%, extra virgin olive oil * 5.7%, apple * 3%, micronized grape pomace * 2.8%, carob flour * 2.5%, fennel powder * 2%, cabbage powder * 2%, spinach powder * 2%, barley grass powder * 2%, micronized grape seeds * 1.4%, kiwi powder * 1.4%, olive leaf powder 0.1% *, natural lemon flavor.	32

**Table 2:** Anthropometric characteristics of the study population under study divided according to treatment group. Abbreviations: BMI, body mass index, F, female; M, male.

	Group	N	Mean Value ± SD
Age (years)	A	6	62.2 ± 5.6
	B	5	57.2 ± 3.4
	C	5	65.6 ± 3.6
	D	5	65.8 ± 1.1
Sex (F/M)	A	6	3/3
	B	5	3/2
	C	5	2/3
	D	5	3/2
Weight (kg)	A	6	80.5 ± 13.3
	B	5	76.8 ± 10.9
	C	5	77.4 ± 10.3
	D	5	70.1 ± 6.0
Height (cm)	A	6	168.3 ± 11.7
	B	5	173.0 ± 9.5
	C	5	164.0 ± 15.3
	D	5	160.4 ± 3.3
BMI (kg/m^2^)	A	6	28.3 ± 4.5
	B	5	25.5 ± 1.8
	C	5	28.9 ± 3.0
	D	5	27.3 ± 3.3

**Table 3:** Laboratory parameters of the study population divided according to treatment group. * This parameter is represented as median (minimum-maximum). Abbreviations: HDL cholesterol, high-density lipoprotein cholesterol; LDL cholesterol, low-density lipoprotein cholesterol.

Laboratory Parameters	Group	T0	T1
Hemoglobin * (g/dL)	A	13.5 (12.3–17.9)	13.2 (12.2–18.0)
	B	14.7 (12.7–15.4)	14.9 (12.6–15.5)
	C	13.1 (10.9–14.2)	12.65 (10.8–14.0)
	D	11.1 (11.2–12.1)	11.8 (11.6–12-0)
Creatinine * (mg/dL)	A	1.32 (0.85–2.21)	1.34 (1.02–2.63)
	B	1.43 (1.16–1.78)	1.37 (1.02–2.06)
	C	1.31 (1.15–1.99)	1.39 (1.17–1.75)
	D	0.96 (0.95–1.32)	1.23 (1.09–1.31)
Glomerular Filtration Rate * (mL/min)	A	52.6 (30.0–92.8)	48.1 (24.3–62.7)
	B	50.7 (32.0–60.1)	45.2 (28.8–78.4)
	C	51.0 (30.0–59.5)	50.15 (29.0–55.0)
	D	54.8 (42.2–82.8)	45.8 (42.6–52.9)
Azotemia * (mg/dL)	A	50 (37–55)	54 (48–63)
	B	50 (32–60)	45 (27–78)
	C	54 (43–66)	47 (44–78)
	D	56 (56–80)	79 (76–88)
Sodium * (mg/dL)	A	142 (132–143)	140.5 (139–143)
	B	141 (139–141)	140 (139–142)
	C	141 (138–143)	140.5 (138–143)
	D	141 (140–141)	143 (141–145)
Potassium * (mg/dL)	A	4.5 (3.8–4.7)	4.4 (3.4–5.0)
	B	4.4 (3.6–4.8)	4.2 (3.8–4.6)
	C	4.3 (3.9–5.1)	4.3 (3.7–4.9)
	D	4.9 (4.7–5.2)	4.8 (4.7–5.7)
Calcium * (mg/dL)	A	9.2 (8.9–10.0)	9.2 (8.7–9.6)
	B	9.6 (9.0–10.3)	9.9 (8.9–9.9)
	C	9.1 (9.1–9.4)	9.1 (8.9–9.7)
	D	9.4 (9.2–9.7)	10.2 (9.0–10.4)
Phosphorus * (mg/dL)	A	3.35 (2.6–4.2)	3.1 (2.9–3.7)
	B	3.2 (3.2–4.0)	3.5 (2.3–3.9)
	C	3.2 (2.6–3.9)	3.2 (2.3–3.9)
	D	3.9 (3.7–4.1)	4.2 (3.9–4.7)
Total cholesterol * (mg/dL)	A	196 (188–240)	170 (153–215)
	B	245(230–333)	200(195–234)
	C	163 (143–192)	163.5 (140–186)
	D	215 (212–218)	220 (210–222)
HDL cholesterol * (mg/dL)	A	48.5 (35–73)	45 (32–66)
	B	58 (52–75)	52 (47–73)
	C	48 (27–67)	46 (27–58)
	D	62 (56–63)	61 (54–64)
LDL cholesterol * (mg/dL)	A	123 (82–175)	115 (97–129)
	B	152 (118–243)	134 (127–218)
	C	82 (67–120)	104 (93–118)
	D	107 (105–122)	130 (129–131)
Triglycerides * (mg/dL)	A	111 (56–275)	92 (48–306)
	B	147 (92–222)	126 (106–159)
	C	115 (45–182)	120 (87–221)
	D	124 (121–127)	118 (110–126)
Uricemia * (mg/dL)	A	6.1 (5.5–7.3)	6.1 (6.4–7.5)
	B	6.6 (5.9–8.6)	6.1 (3.9–7.3)
	C	5.1 (4.3–7.5)	5.8 (4.4–6.9)
	D	7.2 (5.2–7.7)	9.0 (4.5–9.2)
microalbuminuria on a spot morning urine * (mg/g creatinine)	A	14 (4–314)	20 (0–312)
	B	8 (0–240)	11 (0–163)
	C	57 (0–185)	29 (0–80)
	D	12 (10–148)	12(10–92)

**Table 4:** Blood pressure parameters of the study population divided according to treatment group. * This parameter is represented as median (minimum-maximum).

Blood Pressure Parameters	Group	T0	T1
Systolic blood pressure * (mmHg)	A	132 (124–175)	128 (116–154)
	B	138 (126–143)	132 (112–170)
	C	127 (122–159)	128 (122–134)
	D	140 (110–145)	144 (105–148)
Diastolic blood pressure * (mmHg)	A	89 (77–118)	72 (65–95)
	B	89 (82–100)	80 (64–85)
	C	77 (70–91)	77(72–84)
	D	72 (69–80)	70 (71–75)

**Table 5:** Inflammatory and oxidative stress laboratory biomarkers of the study population divided according to treatment group. * This parameter is represented as median (minimum-maximum). Abbreviations: CRP, C-reactive protein; ESR, erythrocyte sedimentation rate; FORD, Free oxygen radical defense; FORT, Free oxygen radical test; OS, oxidative stress.

Inflammatory and OS Biomarkers	Group	T0	T1
FORT * (U)	A	204 (160–358)	276 (160–600)
	B	217 (160–442)	160 (160–413)
	C	229 (171–392)	385 (160–600)
	D	338 (246–340)	358 (233–346)
FORD * (mmol/L)	A	1.61 (0.87–2.26)	1.17 (0.52–1.72)
	B	1.76 (1.01–2.10)	1.25 (1.09–2.23)
	C	1.08 (0.7–1.3)	1.1 (0.87–1.29)
	D	0.62 (0.63–1.21)	1.2 (0.73–1.3)
CRP * (mg/L)	A	4.25 (0.4–9.5)	2.2 (0.4–7.3)
	B	1.7 (1.2–5.1)	1.9 (0.8–5.6)
	C	1.7 (0.4–5.0)	1.45 (0.6–3.2)
	D	5.4 (0.5–5.8)	4.4 (0.5–4.6)
ESR * (mm/h)	A	25 (5–58)	23 (7–44)
	B	32(12–61)	18 (6–63)
	C	19 (2–32)	19 (3–30)
	D	92 (43–96)	85 (48–88)

**Table 6:** Evaluation of body composition assessment, ultrasound. * This parameter is represented as median (minimum-maximum). Abbreviations: BCM, body cell mass; BMI, body mass index; ECW, extracellular water; FFM, fat free mass; FM, fat mass; QRFT, quadriceps rectus femoris thickness; QVIT, quadriceps vastus intermedius thickness; TBW, total body water.

Parameters Useful to Detect Uremic Sarcopenia	Group	T0	T1
Weight * (kg)	A	74.1 (68.6–98.6)	74 (70.1–99.4)
	B	71.8 (65–92.5)	71.5 (64.8–92.5)
	C	74.7 (57.2–88.6)	76.9 (62.3–87)
	D	71.5 (63.6–74.5)	73.5 (62.5–75)
BMI * (kg/m^2^)	A	28.2 (22.5–35.3)	28.7 (22.9–35.5)
	B	24.6 (23.6–27.8)	24.6 (23.5–27.9)
	C	29 (22.1–33.3)	29 (24.8–33.4)
	D	28.7 (23.6–29.7)	27.8 (23.2–29.8)
Resistance * (Ω)	A	494.5 (376–580)	467 (377–582)
	B	569 (525–649)	484 (442–552)
	C	565 (457–663)	535 (371–557)
	D	490 (488–740)	494 (494–694)
Reactance * (Ω)	A	38 (31–45)	45 (37–62)
	B	51 (40–58)	60 (56–68)
	C	44 (35–49)	39 (38–45)
	D	46 (44–48)	48 (43–47)
Phase angle * (°)	A	4.7 (3.8–5.0)	5.9 (5–6.5)
	B	5.5 (5.1–5.8)	6.1 (5.6–6.6)
	C	4.2 (3.8–5.1)	5.1 (4.5–6)
	D	5.1 (3.7–5.2)	5.3 (3.8–5.4)
TBW * (%)	A	39.7 (32.4–54.2)	40.2 (33.9–55.6)
	B	47.9 (46.7–57.9)	53 (47.3–61.5)
	C	48.5 (43.3–60.2)	56.7 (42.8–68.3)
	D	44.9 (45.6–48.9)	47.3 (47.9–48.3)
ECW * (%)	A	51.6 (49.1–58.5)	46.5 (52.5–43.6)
	B	48 (46.7–50.3)	45.4 (42.9–46.1)
	C	55.6 (47.5–59)	53.1 (45.7–56.7)
	D	50.9 (49.9–59.3)	49.4 (49–58.7)
FM * (%)	A	34.6 (23.1–38.7)	28.3 (18.8–30.2)
	B	36.0 (24.1–37.0)	25.6 (18.4–37.8)
	C	34.1 (18.4–43)	35.3 (19.1–41.9)
	D	35.8 (33.8–38.1)	33.5 (34.5–35.1)
FFM * (%)	A	64.6 (61.9–65.6)	74.7 (68.3–81.2)
	B	65.7 (62.5–74.3)	77.6 (66.2–82.6)
	C	65.9 (57–81.6)	64.7 (58.1–80.9)
	D	65.2 (61.9–66.2)	64.5 (63.9–65.5)
BCM * (%)	A	47.3 (40.2–50.1)	51.3 (46.5–56.1)
	B	54 (51.9–56.8)	51.3 (48.8–52.6)
	C	43.2 (39.7–51.7)	45.9 (42.1–53.8)
	D	46.2 (39.3–49.2)	49.1 (40–50.1)
QRFT left * (cm)	A	1.03 (0.89–1.63)	1.12 (0.88–1.98)
	B	1.52 (1.40–1.8)	1.87 (1.26–2.19)
	C	1.23 (0.73–1.8)	1.25 (0.7–1.85)
	D	1.27 (1.34–1.87)	1.34 (1.2–1.84)
QRFT right * (cm)	A	1.05 (0.77–1.74)	1.13 (0.94–1.8)
	B	1.24 (1.05–1.84)	1.50 (1.17–1.90)
	C	1.18 (0.87–1.44)	1.22 (0.7–1.68)
	D	1.65 (1.15–1.7)	1.72 (1.15–1.92)
QVIT left * (cm)	A	0.75 (0.57–1.38)	1.06 (0.72–1.52)
	B	1.60 (1.02–1.90)	1.78 (1.33–2.38)
	C	0.86 (0.76–1.4)	1.01 (0.73–1.54)
	D	1.36 (0.84–1.46)	1.43 (1.00–1.53)
QVIT right * (cm)	A	0.76 (0.62–1.37)	1.14 (0.85–1.46)
	B	1.37 (0.91–2.11)	1.53 (0.98–1.73)
	C	0.87 (0.66–1.64)	1.06 (0.78–1.46)
	D	1.57 (0.87–1.67)	1.47 (0.82–1.67)
Handgrip left * (kg)	A	33.1 (15.2–56.9)	35.2 (16.7–54.0)
	B	42.4 (18.9–55.7)	41.3 (24.3–58.4)
	C	25.4 (14–57.5)	34.3 (18.3–54.3)
	D	26.5 (18.9–27.5)	23.8 (19.1–24.8)
Handgrip right * (kg)	A	39.7 (18.5–56.0)	38.6 (10.2–62.3)
	B	47.9 (26.1–62.3)	44.7 (25.3–62.3)
	C	24.4 (15.6–47.8)	31.3 (17.2–47.5)
	D	23.6 (23.6–23.9)	22.7 (21.7–25.3)

## Data Availability

Data available on request due to privacy restrictions. The data presented in this study are available on request from the corresponding author.

## Abbreviation

% AA	Percentage antiradical activity
AH	Arterial hypertension
APA	Adapted physical activity
BCMI	Body cell mass index
BIA	Bioelectrical impedance analysis
BMI	Body mass index
CDNCDs	Chronic degenerative non-communicable diseases
CKD	Chronic kidney disease
CRP	C-reactive protein
CV	Cardiovascular
ECG	Electrocardiogram
ECW	Extracellular water
EFSA	European food safety authority
ESR	Erythrocyte sedimentation rate
ESRD	End stage-renal disease
EVOO	Extra virgin olive oil
EWGSOP2	European Working Group on Sarcopenia in Older People
FFM	Fat free mass
FM	Fat mass
FORD	Free oxygen radical defense
FORT	Free oxygen radical test
FOXO1	Forkhead box protein O1
GAE	Gallic acid
HGS	Handgrip strength
HPLC-DAD-MS	High-performance liquid chromatography-diode array detector-mass spectrometry
ICW	Intracellular water
IGF-1	Insulin-like growth factor-1
IL-6	Interleukin-6
KD	Kidney disease
KDIGO	Kidney disease: improving global outcomes
LDL	low-density lipoprotein
MD	Mediterranean diet
MM	Muscle mass
MPCs	Minor polar compounds
NF-κB	nuclear factor kappa-light-chain-enhancer of activated B cells
PA	Physical activity
PREDIMED	Prevención con Dieta Mediterránea
QOL	Quality of life
QRFT	Quadriceps rectus femoris thickness
QVIT	Quadriceps vastus intermedius thickness
RAAS	Renin-angiotensin-aldosterone system
RRT	Renal replacement therapy
SCPT	Stair climb power
SF-36	Short form-36
SMWT	Six-minute walk
SOCS-3	Cytokine signalling 3
SPPB	Short physical performance battery
TBW	Total body water
TGF-β	Transforming growth factor-β
TNF-α	Tumor necrosis factor-α
UPP	Ubiquitin-proteasome pathway
US	Uremic sarcopenia
